# Bridging Subjective and Biological Perspectives: Applying an AI-Driven EEG Index in Depression Care

**DOI:** 10.1192/j.eurpsy.2026.11865

**Published:** 2026-07-31

**Authors:** C. M. Chu, H.-A. Chang

**Affiliations:** 1 Psychiatry, Kaohsiung Veterans General Hospital, Kaohsiung; 2 Psychiatry, Tri-Service General Hospital, National Defense Medical University, Taipei, Taiwan

## Abstract

**Introduction:**

Depression often remains underrecognized in Asian contexts due to stigma, limited illness insight, and reliance on self-reports. Objective neurophysiological tools can improve diagnostic clarity. The Stress Electroencephalography (EEG) Assessment (SEA) System™ is an AI-based, TFDA-approved device that analyzes 90 seconds of resting EEG to produce a SEA Index ranging from 1 to 10, where higher scores indicate greater likelihood of major depressive disorder (MDD); values ≥7 are considered positive for depression risk.

**Objectives:**

This report illustrates how integrating the SEA System can strengthen diagnostic confidence, communication, and shared decision-making in MDD through three representative clinical cases.

**Methods:**

Three patients with varied presentations underwent SEA assessment alongside psychiatric evaluation. Each case was analyzed for the SEA Index result and its influence on clinical management.

**Results:**

In Case 1, a student’s high SEA score (9, positive range) resolved parental disbelief and enabled early combined therapy. In Case 2, an academic skeptical of her diagnosis accepted treatment after her elevated score (8) validated MDD risk. In Case 3, a patient reporting remission showed persistent SEA elevation (9), revealing residual symptoms; after medication adjustment, his score dropped to 2 (negative range) with marked improvement. SEA scores thus provided quantifiable neurophysiological evidence supporting diagnostic and therapeutic decisions.

**Conclusions:**

The SEA System offers a rapid, interpretable biomarker of depression severity that complements psychiatric evaluation, reframes depression as a measurable brain disorder, and promotes early detection, stigma reduction, and personalized care.

**Disclosure of Interest:**

C. Chu: None Declared, H.-A. Chang Grant / Research support from: This work was supported by the National Science and Technology Council of Taiwan (NSTC-112- 2314-B-016-017-MY3).

